# Single-Neuron Level One-Photon Voltage Imaging With Sparsely Targeted Genetically Encoded Voltage Indicators

**DOI:** 10.3389/fncel.2019.00039

**Published:** 2019-02-14

**Authors:** Peter Quicke, Chenchen Song, Eric J. McKimm, Milena M. Milosevic, Carmel L. Howe, Mark Neil, Simon R. Schultz, Srdjan D. Antic, Amanda J. Foust, Thomas Knöpfel

**Affiliations:** ^1^Department of Bioengineering, Imperial College London, London, United Kingdom; ^2^Department of Medicine, Imperial College London, London, United Kingdom; ^3^Centre for Neurotechnology, Imperial College London, London, United Kingdom; ^4^Institute for Systems Genomics, Stem Cell Institute, UConn Health, Farmington, CT, United States; ^5^Department of Physics, Imperial College London, London, United Kingdom

**Keywords:** voltage imaging, cerebral cortex, sparse expression, optogenetics, transgenic

## Abstract

Voltage imaging of many neurons simultaneously at single-cell resolution is hampered by the difficulty of detecting small voltage signals from overlapping neuronal processes in neural tissue. Recent advances in genetically encoded voltage indicator (GEVI) imaging have shown single-cell resolution optical voltage recordings in intact tissue through imaging naturally sparse cell classes, sparse viral expression, soma restricted expression, advanced optical systems, or a combination of these. Widespread sparse and strong transgenic GEVI expression would enable straightforward optical access to a densely occurring cell type, such as cortical pyramidal cells. Here we demonstrate that a recently described sparse transgenic expression strategy can enable single-cell resolution voltage imaging of cortical pyramidal cells in intact brain tissue without restricting expression to the soma. We also quantify the functional crosstalk in brain tissue and discuss optimal imaging rates to inform future GEVI experimental design.

## Introduction

Over the last decade, functional fluorescence imaging has become a key technology in cellular and systems neurosciences (Knöpfel et al., 2006; Scanziani and Häusser, 2009; Knöpfel, 2012; Allen et al., 2017; Chen et al., 2017; Otis et al., 2017; Yang and Yuste, 2017). The most prominent applications include *in vivo* imaging of genetically encoded calcium indicators, such as GCaMPs (Nakai et al., 2001; Chen et al., 2013) that have enabled studies of large numbers of single cells longitudinally (Mank et al., 2008; Chen et al., 2012). Imaging of genetically encoded voltage indicators is less widespread despite being a longstanding goal driven by high expectations. The lag between the development of calcium imaging approaches and voltage imaging technologies is because the latter is more demanding due to several intrinsic constraints (Kulkarni and Miller, 2017). Firstly, in contrast to calcium indicators which are localized in the cytosol of the cells of interest, voltage indicators are localized to their plasma membranes, which account for a tiny fraction of their volume. This limits the number of indicator molecules that can be employed and hence the flux of signaling photons that can be generated. Secondly, voltage signals of interest are typically much faster than the signals provided by calcium indicators and therefore must be imaged at higher frame rates. This is not only an instrumentation challenge but also translates, along with the limited number of dye molecules, into a signal-to-noise ratio (SNR) challenge, as a sufficiently high SNR requires a large number of photons sampled per spatiotemporal bin (e.g., 10,000 photons are required in order for a fluorescence change of 1% to have an SNR of 1).

Optical voltage signals need to be imaged at a frame rate sufficient to resolve the signals of interest (e.g., action potentials or subthreshold fluctuations of membrane voltage). However, the appropriate GEVI imaging sampling rate further depends on the time course of the optical signal generated by the GEVI used. Due to the non-instantaneous kinetics of GEVIs the optical signal is generally low-pass filtered relative to fast voltage signals (i.e., action potentials). Imaging at rates higher than necessary degrades SNR by increasing the proportion of time spent on image readout relative to signal integration and increases accumulated read noise. Increasing frame rates without reducing the SNR also necessitates an increase in illumination intensities, which will increase the bleach rate and reduce the available imaging time. Imaging at frame rates above 100 Hz is also limited by image sensor technology and often requires using fast, low pixel-number charge coupled-device (CCD) cameras, or pixel sub-arrays from most modern sCMOS cameras.

When imaging with wide-field illumination, photons are integrated throughout virtually the whole frame period, and therefore fast fluorescent transients can be detected even if the imaging rate is below the nominal Nyquist rate of the optical signal. This is because the integration of detected photons over the frame period applies an effective low-pass filter to the collected fluorescence signal. This contrasts with laser scanning illumination techniques where photons are collected for each pixel for only a short fraction of the frame rate, and a frame rate above the Nyquist frequency must be used to ensure detection of fast transients. That is, a neuron's action potential may escape detection if it occurs between visits of the neuron by the laser spot in LSM. The choice of wide-field imaging speed appropriate to the indicator and experimental question is therefore important.

Improvement of SNR has been the driving force for much of the previous work on voltage imaging. Recently, new photostable low molecular weight voltage sensitive dyes and genetically encoded voltage indicators (GEVIs) have been developed (Gong et al., 2015; Sepehri Rad et al., 2017; Xu et al., 2017, 2018; Abdelfattah et al., 2018; Adam et al., 2018; Chavarha et al., 2018; Piatkevich et al., 2018; Yi et al., 2018). These have greatly increased sensitivity (fluorescence change with change in membrane voltage), considerably increasing achievable SNRs.

Another feature of voltage indicators adds a third issue that needs to be resolved: as a consequence of their plasma membrane localization, optical voltage signals from adjacent cells cannot be resolved without imaging at sub-micrometer resolution, which is largely impractical for functional fluorescence imaging across multiple neurons. Calcium signals, in contrast, are more easily resolved in intact brain tissue as “blinking” cell bodies that can readily be segregated. Hence, alongside low SNRs, allocation of optical voltage signals to individual cells in intact brain tissue is an inherent problem for voltage imaging.

Formally, the issue of single cell resolution can be described as follows: an optical signal from a cell of interest is compromised by shot noise generated by non-signaling fluorescence emanating from the membranes of other fluorescent cells and tissue autofluorescence (the “background”). The fractional change in collected fluorescence, ΔF/F, will be reduced to (1 − *f*_*b*_)Δ*F*/*F* where *f*_*b*_ is the fraction of fluorescence arising from non-signaling structures. Background fluorescence also has a detrimental effect on SNR. In a shot noise limited imaging system, SNR will be reduced proportionally to the SNR measured in the absence of background fluorescence (SNR_0_) as $\sqrt{\left(1-f_{b}\right)}$*SNR*_0_ (Knöpfel et al., 2006). Reducing the excitation volume in an attempt to minimize the contribution of fluorescent membranes of adjacent cells and their processes, for instance by using highly localized two-photon laser scanning (2PLS) excitation, reduces the amount of non-signaling fluorescence collected at the cost of very low rates of signal-carrying fluorescence excitation resulting in low SNRs. This makes 2PLS microscopy a poor choice for most voltage imaging applications, although it has been used successfully in some experimental paradigms (Ahrens et al., 2012; Akemann et al., 2013; Chamberland et al., 2017; Chavarha et al., 2018).

In voltage imaging applications aimed at single-cell resolution, instead of limiting fluorescence excitation to small volumes, a practical approach to maximize the contribution of a single cell to the fluorescence measured across an ROI is to limit the spatial overlap of fluorescence from different cells. Targeting a voltage indicator to single or multiple spatially segregated cells has been achieved by intracellular injection or electroporation of low molecular weight dyes (Antic, 2003; Roome and Kuhn, 2018). This approach, however, is limited to a single or a few cells in the microscopic field of view (FOV). Therefore, voltage imaging based on low molecular weight voltage indicators is practically limited to single dye-injected cells or, using unselective staining procedures, to population imaging without single-cell resolution (Grinvald et al., 2003; Antic et al., 2016). We reasoned that sparse labeling of neurons using genetic methods applicable to GEVIs could provide a practical intermediate experimental paradigm allowing multi-cell voltage imaging at single cell resolution (Song et al., 2017). Sparse labeling has the advantage of the single cell labeling approach of reducing or eliminating unwanted background fluorescence, whilst still labeling multiple cells in a FOV. Sparse labeling neurons will create opportunities for multi-cell, single cell resolution imaging aimed toward understanding signal processing in neuronal networks.

**Figure 1:**
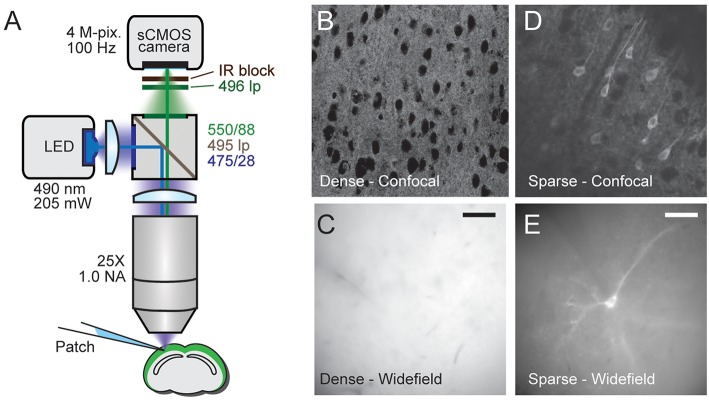
Comparison of densely and sparsely expressed GEVIs. **(A)** The microscope set up for wide-field imaging. We used a standard epifluorescence microscope configuration to image patched cells expressing GEVIs in sparsely and densely expressing brain slices. **(B)** A confocal image of a brain slice densely expressing chimeric VSFP-butterfly. Assignment of fluorescence to individual neurons is impossible due to expression in overlapping membranes. **(C)** A wide-field image of a brain slice with the same expression strategy. The problem of assignment is compounded by the lack of optical sectioning, making even cell bodies difficult to discern. **(D)** A confocal image of a brain slice sparsely expressing VSFP butterfly via destabilized-Cre modulated expression. Processes and soma from individual GEVI-expressing cells can be clearly resolved. **(E)** Wide-field image of a brain slice with the same expression strategy. Contrast is decreased due to lack of optical sectioning, but single cells and processes can still be resolved. Scale bar 40 μm.

Single-cell resolution genetically encoded voltage indicator (GEVI) imaging requires not only sparse, but also strong expression to enable an adequate collected photon flux. Gene delivery via intracortical injection of viruses produces expression patterns where the expression strength and likelihood decreases with increasing distance from the injection site and virus titer. Sparse expression can therefore be achieved with modifications to injection protocols, albeit in a local and highly variable way and with low expression levels (on average < 1 virus particle per cell and therefore often only 1 virus per expressing cell). Another strategy to limit the density of indicator expressing membranes is the targeting of a GEVI to specific compartments of the cell. Recent works showed that, indeed, soma targeting of GEVIs can reduce the number of labeled membranes and enable single-cell signals to be extracted from sequences of images (Abdelfattah et al., 2018; Adam et al., 2018). Voltage imaging of naturally sparse cell populations, such as cortical interneurons, at single cell resolution is achievable with standard transgenic mouse lines (Bayguinov et al., 2017). Dual component expression strategies based on Cre-lox (Sauer and Henderson, 1988) and tetracycline response elements (TRE) (Gossen and Bujard, 1992) are especially useful for GEVI imaging experiments aimed at cortical circuit analyses, as these expression strategies label all cells of a genetic class with high expression levels (Madisen et al., 2015). In the case of non-sparse cell classes, in particular cortical pyramidal cells, sparse stochastic targeting can be achieved by controlling activation of a strong expression cassette via the titratable activity of destabilized Cre (Sando et al., 2013; Song et al., 2017). We previously demonstrated this strategy at the morphological level and demonstrated sparse but strong (“Golgi-staining like”) expression from a GEVI in a transgenic mouse line (Song et al., 2017). Here we validate this approach for functional voltage imaging which requires higher expression levels than simple anatomical imaging. We show that d-Cre based stochastic expression strategies enables single-cell resolution GEVI imaging in cortical pyramidal cells in acute mouse brain slices. We compare recordings from tissue of mice sparsely expressing in cortical pyramidal cells (“sparse PC line”) with recordings from mice which express the GEVI in all pyramidal cells (“pan PC line”) using wide-field imaging (Figure 1A). We evaluate the achievable SNR, discuss optimal GEVI imaging frame rates and also quantify the functional spread of signals to guide experimenters' choice of the sparsity level. We used early generation GEVIs [VSFP Butterfly 1.2 (Akemann et al., 2012) and chimeric VSFP Butterfly YR (Mishina et al., 2014)] for which transgenic mice suitable for a fully genetic approach were readily available. Our principal conclusions should hold true, and quantifications could be extrapolated, for all recently developed GEVIs covering a spectrum of voltage sensitivities, temporal dynamics, and brightness.

## Materials and Methods

### Animals

**Figure 2:**
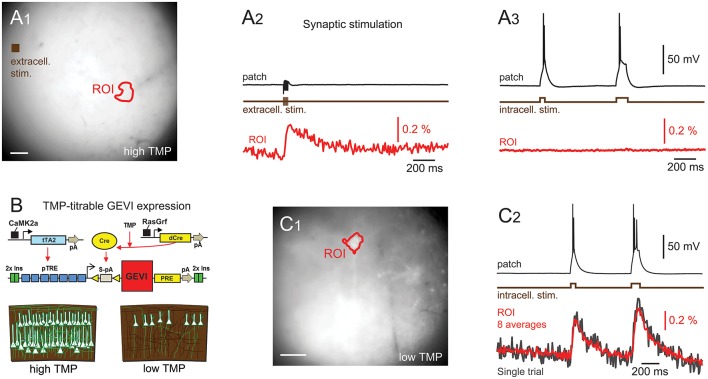
d-Cre modulated sparse expression strategies enable single-cell resolution functional imaging. **(A)** Population, but not single-cell, voltage signals are resolved with densely expressed GEVIs. **(A**_**1**_**)** A wide-field image showing the patched cell's location and the ROI used to generate the voltage imaging trace. An extracellular stimulation electrode was located as indicated. **(A**_**2**_**)** A train of extracellular stimulation evoked responses from many cells, leading to a clear population voltage response in the cellular ROI. **(A**_**3**_**)** No single cell voltage signal could be resolved despite averaging of 80 repeated trials of extracellular stimulation. **(B)** The expression strategy used to generate sparse expression in layer 2/3 pyramidal cells. TMP stabilizes destabilized Cre recombinase enabling TRE driven GEVI expression. **(C**_**1**_**)** A wide-field image of sparsely expressed GEVI showing the ROI used to calculate the voltage time course. **(C**_**2**_**)** Single-cell voltage transients can be clearly resolved with sparse expression. Red time course shows an average of eight repeats, gray shows single trial response. Fluorescence traces shown on inverted *y* axes.

This study was carried out in accordance with the recommendations of UK Animals (Scientific Procedures) Act 1986 under Home Office Project and Personal Licenses (project licenses 70/7818 and 70/9095). The protocol was approved by the UK Home Office. Transgenic mice were bred to express VSFP Butterfly 1.2 in cortical layer 2/3 pyramidal neurons under the intersectional control of TetO and Cre-recombinase [Figure 2B, “sparse PC line,” Rasgrf2-dCre; CaMK2A-tTA; Ai78 (Mayford et al., 1996; Harris et al., 2014; Madisen et al., 2015)]. To induce sparse expression through stochastic re-stabilization of destabilized Cre, a titrated total dose of 2E-4 mg/kg Trimethoprim (TMP, Sigma) was given via multiple intra-peritoneal injections over 2 consecutive days as described in Song et al. (2017). For comparison, transgenic mice densely expressing chimeric VSFP-Butterfly in all pyramidal cells were used [“pan PC line,” CaMK2A-tTA; tetO-chiVSFP (Song et al., 2018)].

### Slice Preparation

Slices from transgenic mice were prepared at least 2 weeks post TMP injection. 400 μm coronal slices were cut using a Campden Microtome 7000 from 4 mice between 50 and 108 days old in ice cold ACSF oxygenated with 95% O_2_/5% CO_2_ containing (in mM): 125 NaCl, 25 NaHCO_3_, 20 glucose, 2.5 KCl, 1.25 NaH_2_PO_4_, 2 MgCl_2_, 2 CaCl_2_. The slices were immediately transferred into NMDG-ACSF (Ting et al., 2014) containing: (in mM) 110 N-Methyl-D glucamine, 2.5 KCl, 1.2 NaH_2_PO_4_, 25 NaHCO_3_, 25 Glucose, 10 MgCl_2_, 0.5 CaCl_2_, adjusted to 300–310 mOsm/kg, pH 7.3–7.4 with HCl, oxygenated with 95% O_2_/5% CO_2_ at 36°C for <12 min before they were transferred back into the original sodium-containing ACSF for at least an hour rest before imaging.

### Imaging

Healthy fluorescent cells were identified using gradient contrast IR and fluorescence optics. Patch pipettes were pulled to a resistance between 3 and 10 MΩ when filled with the following intracellular solution (in mM): 130 K-Gluconate, 7 KCl, 4 ATP—Mg, 0.3 GTP—Na, 10 Phosphocreatine—Na, 10 HEPES. Cells were patched using a Multiclamp 700B amplifier and signals were digitized using a Power 1401 digitizer. Current pulses were injected to elicit action potentials and fluorescence was imaged at 50−100 Hz using a custom-built epifluorescence microscope (optical path shown in Figure 1A).

We excited donor fluorescence of the VSFP FRET fluorescent protein pair with a 490 nm LED (M490L4, Thorlabs) powered by a current driver (Keithley Sourcemeter 1401), collimated with an f = 16 mm aspheric lens (ACL25416U0-A, Thorlabs) and filtered with a 475/28 nm excitation filter (FITC-EX01-CLIN-25, Semrock). Intensity at the sample was between 4 and 30 mW/mm^2^. Fluorescence was collected using a 495 nm long pass dichroic (FF495-Di03, Semrock) along with a 550/88 nm collection filter (FF01-550/88, Semrock), 496 long pass filter (Semrock FF01-496/LP) and IR blocking filter (Semrock, FF01-750/SP) onto a sCMOS camera (512 × 512 pixels with 4 × 4 binning, Orca Flash 4 V2, Hamamatsu). This collected fluorescence from the FRET pair donor fluorophore (mCitrine), meaning membrane depolarizations resulted in decreased fluorescence emission as FRET efficiency increased. Imaging data were acquired using Micromanager (Edelstein et al., 2014). Images recorded at room temperature.

For 500 Hz voltage imaging, a water immersion LUMPlanFl 40 × objective with NA 0.8 (Olympus, Japan) was used. Fluorescence was excited with CoolLED, UK, 473 nm. Optical filters were purchased from Chroma Technology (Rockingham, VT, USA). The filter cube contained an exciter 480/40, dichroic 510DRLP, and emitter 535/50 nm. Voltage signals were sampled at 500 Hz with a NeuroCCD camera (80 × 80 pixel configuration) (RedShirtImaging, Decatur, GA). Analysis of optical data, including spatial averaging, high-pass and low-pass filtering, was conducted with Neuroplex v. 8.0.0 (RedShirtImaging). Images were recorded at 34 C.

### Image Analysis

Imaging trials were repeated between 5 and 20 times and the individual trial image sequences averaged. Linear fits, f_i_, were calculated for each pixel, p_i_, to control for bleaching and the ΔF/F_0_ time course was calculated as (p_i_[n] – f_i_[n])/(f_i_[n] – 1, 600), where 1,600 is the camera offset for 4 × 4 binning and n is the frame number. Only averaged traces were analyzed and all traces plotted are averages unless explicitly noted. Number of averages for traces are noted in captions. We plot all fluorescence traces on inverted y axes.

Image analysis was conducted with Python. Activation maps were found by considering ΔF image sequences calculated pixel-wise as (p_i_[n] – f_i_[n]). The image time series was downsampled to 50 Hz by averaging to improve the SNR. To create maps for visualization and segmentation the image sequences were spatiotemporally filtered with a Gaussian filter with sigma of 1 time point/1 pixel 20 ms/1.04 μm and for functional spread characterization they were temporally filtered with a 3 point median filter. 2D activation maps were then found from 3D image sequences by summing the frames collected during stimulation periods.

ROIs for time series analysis were found by segmenting the activity maps. Each map was thresholded using an automatic criterion (Yen et al., 1995), before a single round of binary closing and then dilation. The largest connected component was then selected as the somatic ROI.

For trials where a long depolarizing stimulus was used the signal size was calculated as the 5th percentile value of the fluorescence time course during the stimulus period with the median value of the previous 10 time points subtracted. For single spike trials the signal size of the first spike in a spike train was calculated differently to the others due to the easier estimation of the fluorescence value immediately preceding. The first spike signal size was calculated as the maximum value in the 50 ms after the stimulus (the stimulus period) with the median value of the preceding 10 time points subtracted. For subsequent spikes in the spike train the signal size was calculated as the minimum of the 50 ms following the stimulus with the maximum of the 20 ms time points before the stimulus subtracted. The noise level of traces was calculated as the standard deviation of 20 samples before the stimulus was applied.

#### Frame Rate Analysis

To examine the effect of decreased sampling rate on GEVI traces we took an example ΔF/F_0_ trace acquired at 500 Hz and downsampled it by averaging. To avoid overestimating the noise level for larger averaging periods we first flattened the trace to remove bleaching remnants by dividing it by a polynomial fit to the trace with the voltage signals removed. We then calculated *n* downsampled traces for downsampling by integer factors of 1, 2, 3, 4, 5, 10, and 20, where each of the *n* traces corresponds to a different downsampling phase. We calculated the noise level as the standard deviation of 100/*n* points with no voltage signal and the signal level as the peak value during the stimulus period subtracted by the median value of the 20/*n* preceding points. AP timing detection jitter was calculated as the imaging frame period multiplied by the range in frame number of the location of the optical AP signal peak. Least-squares log-log-linear fits were then calculated for the noise and 1-signal size.

#### Signal Spread Analysis

We estimated the sparsity level required to separate signals between adjacent cells using the mean autocorrelation of the activation maps. Autocorrelations of spatially unfiltered maps were calculated and a 1-pixel central peak arising from noise in the image was removed by replacing the central pixel with the mean of the surrounding pixels. The autocorrelation images were then normalized to between 0 and 1, rotated such that their longest axis faced the same direction before taking an average over all cells. This generated a roughly elliptical autocorrelation image with orthogonal axes representing our estimate of the largest and smallest signal mixing lengths. We calculated the amplitude of the crosstalk for a neuron at the origin due to other neurons at positions ***r***_*i*_ to be equal to $1-\;\sqrt{\frac{P}{P_{T}}}\sim \;1-\;\sqrt{1/(1+2\underset{i}{\sum }ACF(r_{i}))\;}$ (see Supplementary Methods). We can therefore use the longest and shortest axis of our measured ACF to estimate the best and worst-case mixing fraction, respectively.

## Results

### Voltage Imaging With Densely and Sparsely Targeted GEVIs

As explained in the introduction, it is difficult to optically resolve fluorescent plasma membranes if a GEVI is targeted to all cells in a dense population of neurons, such as cortical pyramidal cells. To illustrate this, Figure 1 shows a comparison of images obtained from two transgenic mouse models using confocal and wide-field microscopy (Figure 1A). In the first mouse line, the GEVI is targeted to all cortical pyramidal cells (“pan PC line,” see Methods). In the second line of mice, the GEVI is targeted to a small subset of (mainly) layer 2/3 pyramidal cells [“sparse PC line” (Harris et al., 2014)]. In the case of the non-sparsely targeted tissue (pan PC), confocal microscopy resolves individual cells bodies by negative contrast of their non-fluorescent cytosol (Figure 1B). Plasma membranes of adjacent cells cannot be differentiated and the bulk of fluorescence emerges from neuronal processes (dendrites and axons) that are not structurally resolved (Figure 1B). Wide-field microscopy of this tissue (pan PC) reveals a sea of fluorescence with virtually no cellular structural details (Figure 1C). In contrast, images of cortical tissue from the sparse PC line resolve the plasma membranes of cell bodies and processes both in confocal (Figure 1D) and in wide-field microscopy (Figure 1E).

To demonstrate that optical voltage signals can readily be recorded in slices from pan PC mice using wide-field imaging, we employed synaptic activation of a large number of optically unresolved neurons (Figure 2A_1_). Optical voltage signals were resolved in single trials over a cell body sized region of interest (ROI) in response to extracellular electrical stimulation (5, 0.5 ms pulses at 100 Hz, electrode ~360 μm from cell body, Figure 2A_2_). This experiment demonstrates that population voltage signals can be recorded across “cell body sized” ROIs in the brain slices of pan PC mice. We then tested whether the optical signal of a single cell can be resolved in the same preparation using wide-field imaging. To this end we patched a layer 2/3 cortical pyramidal cell in current clamp mode. Imaging during intracellular injection of positive current pulses (two pulses of 50 and 100 ms duration, current amplitude adjusted to induce spiking) did not reveal an optical signal across the cellular ROI despite a noise floor of only 0.004% ΔF/F_0_ after averaging the images from up to 80 trials (Figure 2A_3_). This suggests that single cell-level optical voltage signals are masked by the large shot noise produced by the non-signaling fluorescence of neighboring cells.

To verify this prediction, we switched to tissue from the sparse PC line (Figure 2B), where single cells can be resolved even with wide-field epi-illumination fluorescence microscopy (Figure 2C_1_). As with the above experiments using pan PC tissue, we patched a single fluorescent cell and imaged while the cell was depolarized by direct current injection through the patch electrode, readily resolving optical voltage signals at the single cell level (Figure 2C_2_).

In response to long lasting depolarizing current injections (50–500 ms, adjusted to induce one or more APs), we resolved voltage signals in single trials (*n* = 12 out of 15 cells attempted). In response to short single spike-triggering current pulses (0.5 ms current injections to induce spiking at 20 Hz), we observed signals (*n* = 4 cells out of 4 cells attempted), with 2 cells with a signal-to-noise ratio consistent with spike detection without trial averaging (see below). Overall, we resolved single cell voltage signals in 13 patched cells in 13 slices at imaging depths of up to 77 μm.

**Figure 3:**
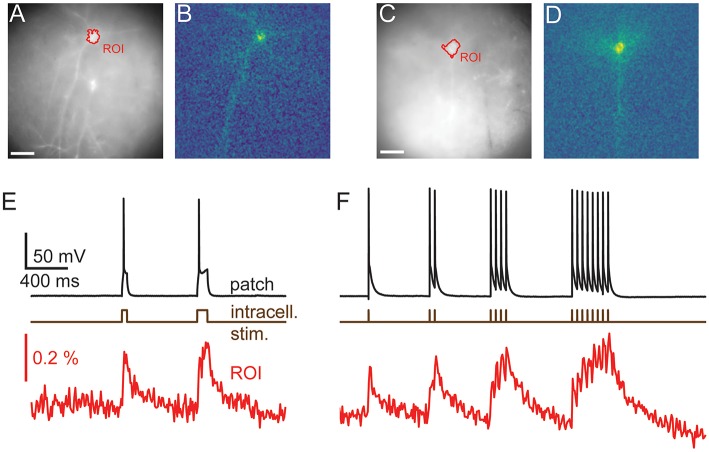
Imaging reveals dendritic structure. Wide-field **(A,C)** and activity maps **(B,D)** of two cortical pyramidal cells. Activity maps were generated by calculating the integral over stimulated video frames and pick out an individual cells dendritic structure, including basal and apical dendrites, from adjacent cells. **(E,F)** the average time courses from the videos used to calculate the activity maps. Optical traces are averages of 12 **(E)** and 10 **(F)** repeats, scale bars 40 μm. Fluorescence traces shown on inverted y axes.

Interestingly, maps of GEVI responses (ΔF images, see Methods) reveal cellular structures that are not always readily apparent in raw wide-field images. Maps of GEVI responses allow identification of processes belonging to active cells (Figure 3). We calculated response maps (Figures 3B,D) from our ΔF image series by averaging 7–30 frames during stimulus periods, depending on the stimulus length. As the GEVI fluorescence decreases on membrane depolarization, pixels containing signal have a negative value in response to the stimulus whilst pixels containing only noise average to zero (see Methods section). The response maps clearly reflect the anatomy of the patched cells; dendrites and their branches can also be seen when they lie in the plane of focus. 6/13 cells were imaged with one or more other cells in the FOV and in these cases the active cell can be clearly distinguished in the activity map from the adjacent GEVI-labeled cells (Figure 3, see also below, Figure 6). We used these activity maps to automatically segment the cell soma from the image sequences by thresholding the activity maps. We then calculated the cell's fluorescence time course as the mean time course of the pixels within the segmented region (Figures 3E,F).

### Signal-To-Noise Ratio

Comparing experimentally assessed SNR values between different imaging systems and preparations is complicated by differing fluorescence collection efficiencies, illumination conditions, and GEVI expression levels. Optical signals collected with high quality sCMOS cameras operating in a high photon count regime [>>10 photons per pixel per frame (Li et al., 2016)] are shot noise limited. With our measured baseline noise levels < 1%, we clearly image in this regime. This means that we can write the SNR as $SNR\;\propto \;\frac{\Delta F}{F}\sqrt{n}$, where ΔF/F is the fractional change in fluorescence, or GEVI sensitivity, and n is the number of collected fluorescence photons. Increasing the illumination intensity increases the number of photons collected, increasing SNR, but in doing so increases the rate of bleaching of the sample, thus reducing the available imaging time. This makes fair GEVI evaluation and selection difficult as data from disparate labs must be collated and compared. A practical way to increase comparability of data from different systems, is to take the rate of photobleaching into account. Bleach rates can be used to normalize across different illumination intensities and facilitate comparison across different GEVIs with different photostabilities. The bleach rate, dC/dt, is proportional to −*I*_*ex*_Φ_*B*_*C*, where I_ex_ is the illumination intensity, Φ_B_ the bleaching quantum yield, and C the concentration of unbleached GEVIs. Ideally the quantum yield of bleaching would be measured for each GEVI as it would allow independent comparison of expression density; however, this is not known for the GEVI used in this study or, to our knowledge, any other GEVI. Normalizing the SNR to the bleach rate, however, can help to control for the GEVIs bleaching propensity and concentration along with the illumination level in one step with an easily calculable number.

**Figure 4:**
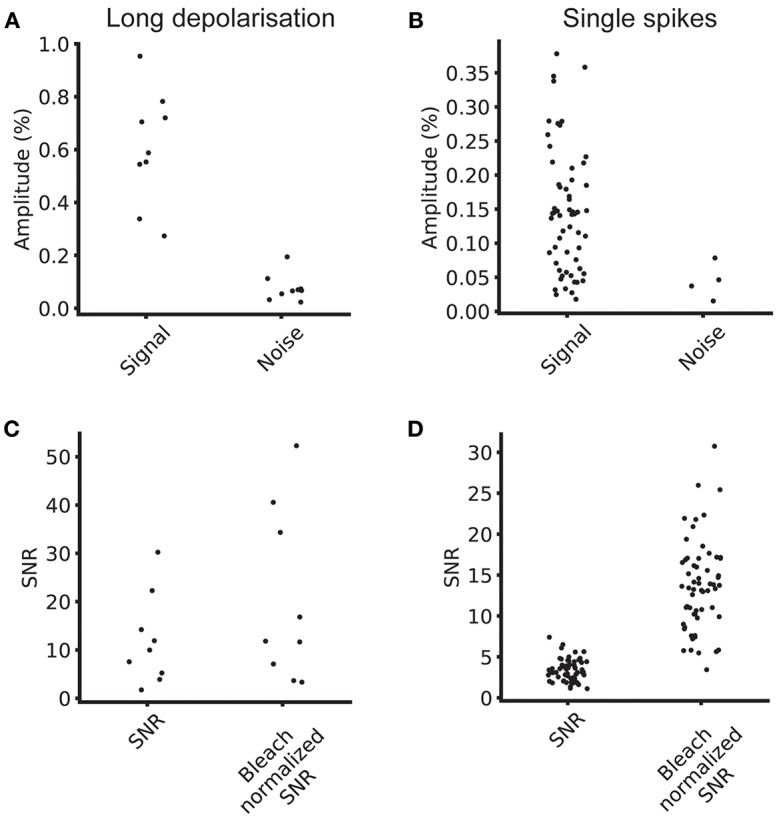
Signal and noise levels. **(A)** Average signal and noise amplitudes for individual cells for five repeats at 100 Hz. **(B)** Single spike signal amplitude and noise amplitude for five repeats at 100 Hz. **(C)** SNR and bleach corrected SNR for long depolarizing stimulus. **(D)** SNR and bleach normalized SNR for single spike stimulus.

To this end, we estimated the signal size, the bleaching rate and the SNR of the optical signals across a segmented ROI. The median bleach rate over the cellular ROIs was 0.52%/s and the 90th and 10th percentiles were 0.98 and 0.23%/s (calculated using a linear fit of baseline fluorescence). We calculated the signal and noise levels for 9/15 cells for which we collected data at 100 Hz with comparable stimulus regimes, and used image sequences of the average of four trials for each cell in the analysis. Table 1 summarizes the signal, noise, SNR and bleach corrected SNR values measured for long depolarizing stimuli (100 ms pulse duration, Figure 3E) and AP inducing stimuli (Figure 3F). The values are reported as median [10th percentile, 90th percentile]. We plot the individual measured values in Figure 4.

**Table 1:** Signal, Noise, and SNR values.

**Stimulus type**	**Signal size (%)**	**Noise level (%)**	**SNR**	**Bleach corrected SNR**	**Number of cells**
100 ms depolarization	0.59 [0.33, 0.82]	0.07 [0.03, 0.13]	10.0 [3.5, 23.9]	11.8 [3.6, 42.4]	9
Action potential	0.14 [0.04, 0.28]	0.04 [0.02, 0.07]	3.1 [1.8, 4.8]	13.5 [7.1, 21.0]	4

These SNR values come from an early generation GEVI with low sensitivity. As SNR increases linearly with indicator sensitivity, our values and conclusions can easily be scaled for more recent GEVIs with much higher reported sensitivity under similar experimental conditions.

### Effect of Sampling Rate

**Figure 5:**
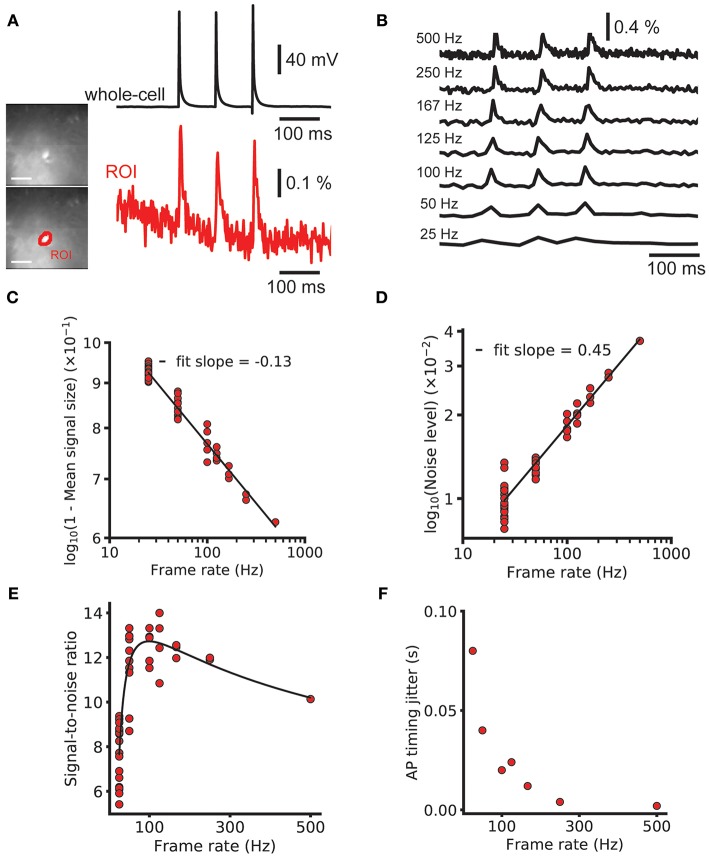
Effect of frame rate on SNR and spike timing estimation. Decreasing the imaging rate in wide-field imaging reduces the temporal accuracy and AP shape information but increases the SNR. **(A)** Image of a brain slice from sparse GEVI mouse obtained at 500 Hz frame rate (80 × 80 pixels). Simultaneous electrical recording from cell body (black) and optical recording (red). Average of nine trials. **(B)** A 500 Hz ΔF/F trace with 3 single-spike transients and down samples by averaging. Signal size, noise level and AP timing estimation accuracy all decrease with mean downsampling. **(C,D)** Power law fits to 1—signal size and the noise level. The noise level scales approximately with the square as expected from Poisson statistics. **(E)** The signal to noise ratio and power law fits plotted on linear axes. Fits give an optimal frame rate for spike detection SNR of 99 Hz. Note that this curve is the result of the division of the log-log linear fits in **(C,D)** and not a fit to the plotted points. **(F)** The bounds on spike timing estimation for different frame rates. Fluorescence traces shown on inverted y axes.

As discussed in the introduction, due to the integration of photons during the whole frame period, slow camera frame rates may be compatible with GEVI-based AP detection, especially for experiments that can tolerate low precision of AP onset timing. To investigate these predictions, we imaged at 500 Hz using a fast, low resolution (80 × 80 pixel) CCD camera (Figure 5A, images). Three brief current pulses (pulse duration = 2 ms, pulse interval = 100 ms) were delivered via a patch pipette to drive a triplet of action potentials (Figure 5A, whole-cell). We examined the peak SNR of AP related signals for a voltage imaging trace imaged at 500 Hz and integer downsamples by *n* of the *n*-point moving average. A fluorescent time course containing 3 AP-evoked fluorescence transients was downsampled by averaging to frequencies as low as 25 Hz (Figure 5B) and the signal and noise were measured (Figures 5C,D). The noise and signal varied with the relative phase of the downsampling. The mean downsampled noise level was well fit by a power law model of the form A_2_x^b2^ (log-log linear *r*^2^ = 0.999), with b_2_ = 0.45 ≈ 0.5 as predicted by theory (Gans and Gill, 1983). The mean downsampled peak signal was also well fit by a power law fit of 1 – A_1_x^b1^ (*r*^2^ = 0.995), with b_1_ = −0.13. Combining these and plotting again the SNR gave a predicted ideal imaging speed for spike detection of 99 Hz (Figure 5E). We note, however, that the ideal imaging speed may be much higher if precise information about AP shape or spike timing (see spike jitter as a function of imaging rate, Figure 5F) is desired and a hypothetical GEVI with very fast kinetics is used.

We validated this analysis by modeling the GEVI signal as a series of decaying exponentials. We calculated the fraction of aliased power compared to total sampled power as a function of sampling frequency when sampling by integrating through the sampling period. We found that severe aliasing occurs when the sampling period used is more than approximately half the decay constant of the optical signal. The signal in Figure 5A was well fit with an exponential decay with a time constant of 12.0 ms. This predicts a frame rate of around 167 Hz is sufficient to avoid significant aliasing. The optical signals recorded at 100 Hz are slower, as they were recorded at room temperature, as opposed to 34 C for the 500 Hz traces. For these, 100 Hz imaging is sufficiently high to prevent significant aliasing.

### Signal Spread

**Figure 6:**
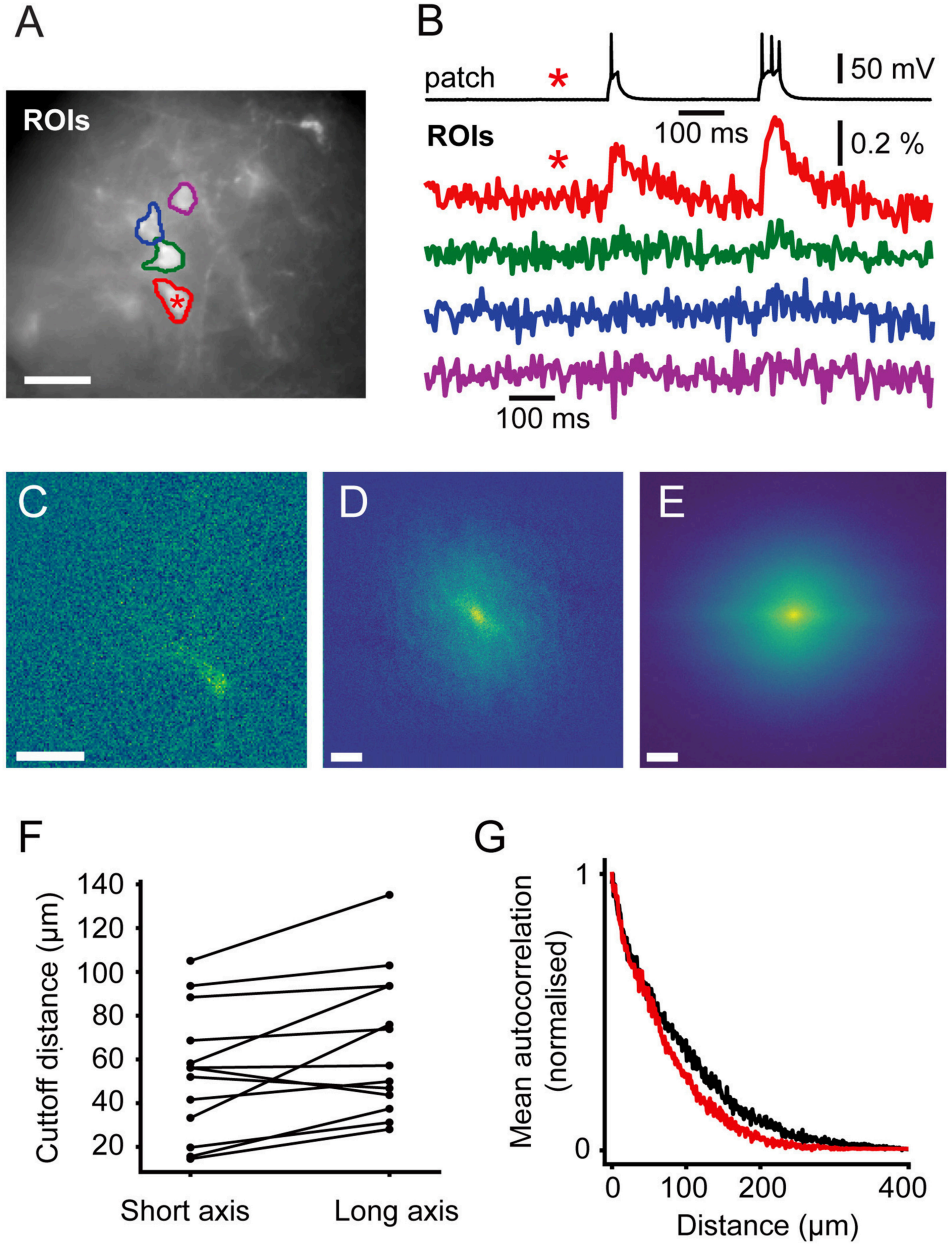
Signal Spread. Autocorrelation of activity maps can be used to estimate crosstalk. **(A)** Wide-field image of a patched neuron (red roi) with other neurons in the FOV. **(B)** Electrophysiological and fluorescent trace from the patched active cell (red and black) and fluorescent traces from ROIs over adjacent cells (green, blue and magenta). A small amount of crosstalk can be seen in the time courses of the adjacent cells. **(C)** A spatially unfiltered activity map of the same neuron calculated by measuring the sum over frames during the depolarizing stimulus. **(D)** The 2D spatial autocorrelation of the activity map. This represents the fraction of signal power arising from the patched neuron at different separations. **(E)** The mean of rotationally aligned autocorrelations of all measured cells. **(F)** The length of the long and short 50% cut-offs for all cells. **(G)** The profile of the mean autocorrelation for the long (black) and short (red) axes. Scale bars 40 μm.

In order to guide future levels of sparsity we developed a method to estimate the level of signal mixing between cells as a function of distance. Neurons are highly variable in shape and, in the cortex, their extent varies with the orientation of the imaging focal plane relative to the cortical column. Figure 6A shows an example ROI containing a patched, stimulated cell adjacent to unpatched cells. The fluorescent voltage response in Figure 6B shows a clear transient in the time course over the patched cell. Fluorescent time courses from ROIs over adjacent unpatched cells show a small amount of crosstalk, disappearing for more distant cells. This indicates that time courses from adjacent cells can be discriminated, albeit with some signal mixing depending on distance.

In order to quantify the expected level of this signal mixing we considered how the time course of one neuron of interest would be affected by being surrounded by *i* other neurons separated by distances, *r*_*i*_, of the same shape and with similar fluorescent time courses. We wanted to quantify crosstalk, that is the signal amplitude (square root of the signal power, P, $\sqrt{P}=\sqrt{\int s^{2}dt}$), arising from adjacent neurons when averaging signal from a ROI over a target neuron as a fraction of the total signal. This crosstalk was calculated (see Supplementary Methods) to be given by $1-\;\sqrt{\frac{P}{P_{T}}}\sim \;1-\;\sqrt{1/(1+2\underset{i}{\sum }ACF(r_{i}))\;}$, where ACF(r) is the spatial autocorrelation function of the neurons' activity (ΔF) map, such as in Figures 3B,D, 6C, evaluated at distance r. P and P_T_ correspond to the signal power from the target neuron and total measure power, respectively. We calculated autocorrelations from the activation maps of signal-containing pixels (Figure 6C), which allowed us to simply average different cells together. We averaged all the autocorrelations together after aligning their longest axes to average out cell-structure specific features caused by different dendritic shapes (Figure 6E). To quantify the amount of crosstalk expected in sparse imaging we first calculated the width for each cell where the autocorrelation fell to 50% of its central value (Figure 6F). This 50% cutoff represents the distance at which the crosstalk it contributes to an adjacent neuron would be 50% of the signal power or 25% of signal amplitude (where signal amplitude is the square root of the signal power). The 50% cut-offs of the neurons recorded ranged from 15 to 135 μm. The large range in the values can be attributed to differences in imaging depth and dendritic structure. We plot the mean falloff in the autocorrelation in Figure 6G. Using this, we calculated the crosstalk for two cells separated by 100 μm the proportion of the total signal amplitude accounted for by the cell of interest when trying to image a single cell as between 20 and 22%. For cells on a hexagonal grid of side length 100 μm the crosstalk was found to be 52–55%.

## Discussion

We have shown that destabilized Cre recombinase (dCre) based expression strategies enable single-cell resolution voltage imaging of cortical pyramidal cells in acute brain slices. This expands the current repertoire of single-cell voltage imaging from interneurons and other naturally sparse cell classes to non-sparse layer 2/3 cortical pyramidal cells. We have not verified this approach with other cell classes, although we expect no differences in the strategy relevant to functional imaging. This powerful approach allows the sparsity of expression to be varied by the titration of the dosage of TMP which stabilizes the dCre and, in combination with strong transgene inducers systems, such as tetO, enables sparse but strong GEVI expression. Different levels of sparsity allow the experimenter to trade off the level of population sampling and functional crosstalk between cellular voltage traces. The level of sparsity can also be adapted to the imaging approach used, with increased specificity from, for example, holographic imaging allowing denser cellular expression (Foust et al., 2015).

Although in this specific implementation, signal averaging was often needed in order to resolve single-AP voltage transients, an issue common to many voltage imaging applications due to low sensitivity of earlier generation GEVIs, such as those used here. However, this genetic approach is, at least conceptually, not restricted to particular GEVIs, and could be used with any of the more sensitive GEVIs that have been recently reported (Abdelfattah et al., 2018; Adam et al., 2018; Piatkevich et al., 2018; Yi et al., 2018). We anticipate that these new GEVIs, combined with the sparse transgenic strategy used in the current study, will yield better single trial spike detection performance.

As wide-field single photon excitation was used, the imaging depth was limited by the scattering of both excitation and emission light. We were able to record from cells at depths up to 77 μm in slice, although at these depths there was severe broadening of the functional signal due to scattering. This scattering will limit wide-field *in vivo* imaging in mice to superficial cortical layers. Two-photon excitation would improve this performance, however, this would require optimizing both the GEVI for two-photon excitation, and also using improved imaging methods to increase traditional two-photon imaging's meager photon budget (Cheng et al., 2011; Prevedel et al., 2016; Chavarha et al., 2018; Quicke et al., 2018; Schuck et al., 2018). Contributing to the difficulties of two-photon voltage imaging, as each pixel is sampled for only a short fraction of the frame rate, full Nyquist-rate sampling of the indicator kinetics must be used to avoid aliasing of high frequency voltage transients.

Soma targeting of voltage indicators, as seen in recent GEVI studies (Abdelfattah et al., 2018; Adam et al., 2018) is likely to greatly reduce the mixing of functional signals by restricting the possible spatial sources of contaminating signal. This comes at the cost, however, of eliminating the ability to reveal somato-dendritic physiology. In multi-site voltage imaging experiments, successful soma targeting will eliminate important clues about input signals generated by dendritic neuropil.

We analyzed how far functional signals from fully labeled (not soma-targeted) active cells spread in order to guide future experimental designs. To choose a specific sparsity level, however, experimenters must also consider other factors, such as the characteristics of the membrane dynamics of the studied cell class, as well as the specific experimental question. More crosstalk can be tolerated, and therefore more dense cells studied, if factors, such as AP amplitude can be used to discriminate between signals from different cells and subthreshold fluctuations are less important.

Sparse transgenic approaches could also be beneficial to opsin photostimulation approaches. Light shaping methods using computer generated holography (Papagiakoumou et al., 2008; Pégard et al., 2017) or generalized phase contrast (Papagiakoumou et al., 2010) can benefit from sparsely expressed opsins to better confine excitation to a single cell or subsets of cells. Previous studies have already used soma-restricted expression (Shemesh et al., 2017) or exploited naturally sparse cell classes (Papagiakoumou et al., 2013; Ronzitti et al., 2017) to improve single-cell targeting with optogenetic actuation. Sparse expression strategies as used here could also help with excitation confinement whilst enabling holographic dendritic stimulation of individual cells (Yang et al., 2011).

## Data Availability Statement

The datasets generated for this study are available on request to the corresponding author.

## Author Contributions

PQ, CS, SA, AF, and TK conceived and designed the experiments. PQ, CS, EM, MM, and CH performed experiments. PQ, SA, MN, SS, AF, and TK designed the analysis. PQ, SA, and TK analyzed the data and wrote the paper. All authors contributed to manuscript revision and approved the final manuscript.

### Conflict of Interest Statement

The authors declare that the research was conducted in the absence of any commercial or financial relationships that could be construed as a potential conflict of interest.
