# Corrigendum: Single-Neuron Level One-Photon Voltage Imaging With Sparsely Targeted Genetically Encoded Voltage Indicators

**DOI:** 10.3389/fncel.2019.00202

**Published:** 2019-05-07

**Authors:** Peter Quicke, Chenchen Song, Eric J. McKimm, Milena M. Milosevic, Carmel L. Howe, Mark Neil, Simon R. Schultz, Srdjan D. Antic, Amanda J. Foust, Thomas Knöpfel

**Affiliations:** ^1^Department of Bioengineering, Imperial College London, London, United Kingdom; ^2^Department of Medicine, Imperial College London, London, United Kingdom; ^3^Centre for Neurotechnology, Imperial College London, London, United Kingdom; ^4^Institute for Systems Genomics, Stem Cell Institute, UConn Health, Farmington, CT, United States; ^5^Department of Physics, Imperial College London, London, United Kingdom

**Keywords:** voltage imaging, cerebral cortex, sparse expression, optogenetics, transgenic

In the original article, there was an error. The in-line equation for the signal-to-noise ratio in the presence of a fluorescence background was incorrectly printed as $\sqrt{\left(1-f_{b}\right)*SNR_{0}}$, where it should have been printed as $\sqrt{\left(1-f_{b}\;\right)}SNR_{0}$.

A correction has been made to the **Introduction**, paragraph six:

“Formally, the issue of single cell resolution can be described as follows: an optical signal from a cell of interest is compromised by shot noise generated by non-signaling fluorescence emanating from the membranes of other fluorescent cells and tissue autofluorescence (the “background”). The fractional change in collected fluorescence, ΔF/F, will be reduced to (1−*f*_*b*_)Δ*F*/*F* where *f*_*b*_ is the fraction of fluorescence arising from non-signaling structures. Background fluorescence also has a detrimental effect on SNR. In a shot noise limited imaging system, SNR will be reduced proportionally to the SNR measured in the absence of background fluorescence (SNR_0_) as $\sqrt{\left(1-f_{b}\right)}SNR_{0}$ (Knöpfel et al., 2006). Reducing the excitation volume in an attempt to minimize the contribution of fluorescent membranes of adjacent cells and their processes, for instance by using highly localized two-photon laser scanning (2PLS) excitation, reduces the amount of non-signaling fluorescence collected at the cost of very low rates of signal-carrying fluorescence excitation resulting in low SNRs. This makes 2PLS microscopy a poor choice for most voltage imaging applications, although it has been used successfully in some experimental paradigms (Ahrens et al., 2012; Akemann et al., 2013; Chamberland et al., 2017; Chavarha et al., 2018).”

The authors apologize for this error and state that this does not change the scientific conclusions of the article in any way. The original article has been updated.
