# Supplementary material for: Single-Neuron Level One-Photon Voltage Imaging With Sparsely Targeted Genetically Encoded Voltage Indicators
Source: Front Cell Neurosci. 2019 Feb 14;13:39. doi: 10.3389/fncel.2019.00039 (PMC6413708; doi:10.3389/fncel.2019.00039)
Supplement: Supplementary file 1 [file Data_Sheet_1.pdf]

1 Crosstalk derivation

Consider two identically shaped neurons separated by a distance d, labelled as 1 and 2. We are interested in measuring the time course of neuron 1 without measuring the signal from neuron 2. The signal containing pixels imaged onto the camera are given by $f_1(x, y, t)$ and $f_2(x, y, t)$. We assume that the neurons are of equal brightness, spatially identical and homogeneous so that we can separate the response functions into $f_1 = f(x, y)p(t)$ and $f_2 = f(x + d, y)q(t)$. We also assume that the functional signal from the neurons decays with distance from the soma. The signal is given by $s = f_1 + f_2$. We wish to calculate the fraction of signal power arising from neuron 1 when integrating over a region of interest (ROI), R, covering the majority of neuron 1. The total signal power arising from the ROI is given by

$$
\begin{aligned}
P_T &= \int_0^\infty \int_R S^2 \, dx \, dy \, dt \\
&= \int_0^\infty \int_R f_1^2 + f_2^2 + 2 f_1 f_2 \, dx \, dy \, dt \\
&= \int_0^\infty p^2(t) \, dt \int_R f^2(x, y) \, dx \, dy + \int_0^\infty q^2(t) \, dt \int_R f^2(x + d, y) \, dx \, dy + \\
&\quad 2 \int_0^\infty p(t)q(t) \, dt \int_R f(x, y) f(x + d, y) \, dx \, dy.
\end{aligned}
$$

If the region R encompasses enough of $f(x)$ then we can approximate the final integral with the 2 dimensional autocorrelation function,

$$
ACF(x, y) = \int_{-\infty}^{\infty} \int_{-\infty}^{\infty} f(x, y) f(x + x', y + y') \, dx' \, dy', \tag{1}
$$

evaluated at the point $(d, 0)$. Voltage imaging temporal signals consist of few-percentage fluctuations on a significant background and we can write them in the form $k_0 + k_1(t)$, where $k_1 << k_0$. To continue with our calculation we assume that the power in the temporal fluctuations of the neuron's time courses is equal and that they are uncorrelated. We can therefore factorise the integral in time as

$$
\int_0^\infty p^2(t) \, dt = \int_0^\infty q^2(t) \, dt = \int_0^\infty p(t)q(t) \, dt = K \tag{2}
$$

and write the total power in region R as

$$
P_T = K \left(S_1 + S_2 + 2 \, ACF(d, 0) \right), \tag{3}
$$

Where $S_1 = \int_R f^2(x, y) \, dx \, dy$ and $S_2 = \int_R f^2(x + d, y) \, dx \, dy$. We wish to calculate the fraction of power arising from neuron 1, given by

$$
\begin{aligned}
\frac{P_1}{P_T} &= \frac{P_1}{K \left(S_1 + S_2 + 2 \, ACF(d, 0) \right)} \\
&= \frac{S_1}{\left(S_1 + S_2 + 2 \, ACF(d, 0) \right)}.
\end{aligned}
$$

If the separation, d, is sufficiently large such that $f(x+d,y)$ is much less than 1 over the region R then $S_2 << 2\,ACF(d,0) << S_1$ and we can approximate the power ratio as

$$\frac{P_1}{P_T} \approx \frac{S_1}{(S_1 + 2\,ACF(d,0))}. \tag{4}$$

S_1 is equal to the autocorrelation function evaluated at the origin and we can therefore normalise this to 1 with no loss of generality:

$$\begin{aligned}\frac{P_1}{P_T} &\approx \frac{ACF(0,0)}{(ACF(0,0) + 2\,ACF(d,0))}\\ &= \frac{1}{(1 + 2\,ACF(d,0))}\end{aligned}$$

Similarly, considering the impact of multiple cells at positions $\vec{r_i}$ we can calculate the fraction of power arising from the target neuron as:

$$\frac{P_1}{P_T} \approx \frac{1}{(1 + 2\sum_i ACF(\vec{r_i}))}. \tag{5}$$
